# Editorial: Ischemic retinopathy: underlying pathologic mechanisms and identifying therapeutic molecular targets

**DOI:** 10.3389/fopht.2025.1707494

**Published:** 2025-09-24

**Authors:** Deokho Lee, Dong Ho Park

**Affiliations:** 1 The Korean Institute of Nutrition, Hallym University, Chuncheon, Republic of Korea; 2 Department of Ophthalmology, Keio University School of Medicine, Tokyo, Japan; 3 Department of Ophthalmology, School of Medicine, Kyungpook National University, Daegu, Republic of Korea; 4 Department of Ophthalmology, Kyungpook National University Hospital, Daegu, Republic of Korea; 5 Cell & Matrix Research Institute, Kyungpook National University, Daegu, Republic of Korea; 6 BK21 FOUR KNU Convergence Educational Program of Biomedical Sciences for Creative Future Talents, Daegu, Republic of Korea

**Keywords:** myopia, night blindness, multiple evanescent white dot syndrome, central retinal artery occlusion, diabetic retinopathy, choroidal blood flow

The retina, one of the metabolically active tissues in our body, is mainly supplied from the central retinal artery as well as the choroidal vascular network. Each metabolic condition in the dual supply system can cause a distinct damage in the retinal layers, ultimately leading to the development and progression of the different eye diseases, such as age-related macular degeneration, glaucoma, or diabetic retinopathy.

In this Research Topic, we summarized various pathophysiologic alterations in the eye and/or find pathologic biological parameters from three original research and one case report articles with different disease conditions in the eye (e.g. myopia, central retinal artery occlusion/CRAO, diabetic retinopathy/DR, and multiple evanescent white dot syndrome/MEWDS).

First of all, Ye et al. aimed to examine the link between axial length to corneal curvature radius ratio (AL/CR, used for evaluating the ocular refractive state) and choroidal blood flow in 202 eyes from myopic children in China. They mainly found that AL/CR is negatively correlated with choroidal blood flow perfusion, implying a crucial link between refractive biological parameters and ocular blood circulation under myopic conditions. The choroidal blood flow changes could be a secondary outcome in myopia development and progression. This also shows that myopia can be considered as an ischemic eye condition that needs attention to ocular microcirculation changes. As the prevalence of myopia in Asia (including China and Republic of Korea) is high and expected to increase more, considering the potent microcirculation issues in myopia patients might be helpful to manage the disease development and further progression.

Sanie-Jahromi et al. tried to find the pathologic effects of hyperglycemia and therapeutic roles of different insulin formulations (including regular, glulisine, and aspart) against DR progression using two human retinal cell types. Eyeballs from an organ donor were used to obtain human retinal endothelial cells (HRECs) and retinal pigment epithelium (RPE) cells. They highlighted the distinct biological effects of insulin analogues (regular, glulisine, and aspart) depending on the cell types, and concluded that aspart insulin might be more useful to modulate diabetes-mediated pathologic molecules in the eye, such as vascular endothelial growth factors and/or angiotensinogen. This comparative insulin study could be used to develop promising therapeutic strategies to help managing the progression of DR along with traditional glycemic control.

Sano et al. attempted to unravel the therapeutic effects of prostaglandin E_1_ (PGE_1_) against vision-threatening CRAO cases. Based on the result from their retrospective study, the PGE_1_-treated group had better best-corrected visual acuity in comparison with the control group, while no dramatic side effects were detected in either group. This intriguing study enables further consideration of PGE_1_ use as a potent treatment for ischemic retinopathy including CRAO.

Miyake et al. reported two cases of MEWDS (a rare inflammatory ocular condition with a potent result of non-perfused choriocapillaris) with transient night blindness in Japan. They found a decrease in rod-dominant amplitude on full-field electroretinography in MEWDS from onset, with symptoms resolving within three months. This shows that outer retinal layer damage in MEWDS could be reversed, consistent with the general feature of MEWDS.

In conclusions, many people suffer from visual impairment and blindness induced by retinal ischemia. Although more studies are needed, from the current Research Topic, we could cover the advanced state of art in various ischemic conditions in the eye (such as, myopia, CRAO, DR, and MEWDS), and further present several promising therapeutic approaches including insulin therapy or PGE_1_ treatment. We hope this Research Topic could improve the ongoing or help future research on developing therapeutics for ischemic retinopathy.

